# Histamine: A Mediator of Intestinal Disorders—A Review

**DOI:** 10.3390/metabo12100895

**Published:** 2022-09-23

**Authors:** Sylwia Smolinska, Ewa Winiarska, Anna Globinska, Marek Jutel

**Affiliations:** 1 Department of Clinical Immunology, Wroclaw Medical University, 50-368 Wroclaw, Poland; 2 Swiss Institute of Allergy and Asthma Research, CH-7265 Davos, Switzerland; 3 “ALL-MED” Medical Research Institute, 53-201 Wroclaw, Poland

**Keywords:** histamine, histamine receptors, histamine intolerance, food allergy, inflammatory bowel disease, irritable bowel syndrome, scombroid poisoning, colorectal cancer

## Abstract

Within the gastrointestinal tract, histamine is present at relatively high concentrations, especially during inflammatory processes. Histamine is a biogenic amine with numerous effects on many cell types, mediated by the activation of its four different histamine receptors (H1–H4Rs). It is produced and released by immune cells as mast cells and basophils. Some cells such as dendritic cells or T cells can express histidine decarboxylase, an enzyme for histamine synthesis after stimulation. The same can be done by the human gut microbiota. The production of histamine by bacteria in the human gut influence the immune response, although the major source of histamine is food. The large spectrum of histamine effects on a number of cellular processes results in various gastrointestinal disorders including food allergy, histamine intolerance, irritable bowel syndrome, and inflammatory bowel disease, among others. In this review, the protective or pathogenic effects of histamine on various gut disorders are discussed.

## 1. Histamine 

Histamine [2-(4-imidazolyl)-ethylamine] is a biogenic amine that was first synthesized in the early 1900s. Since then, its functions have started to be discovered and more well-described [1,2]. Histamine, which is found in many cell types, seems to be the most pleiotropic molecule in the human body [3]. The best known action of histamine is to induce contraction of smooth muscle cells (including bronchi and intestines) as well as dilate blood vessels and increase their permeability. Histamine causes heart rhythm disturbances and influences blood pressure, increases mucous secretion, gastric acid secretion, and irritation of nociceptive nerve fibers [4,5]. Histamine may also play a role in neurotransmission, immunomodulation, hemopoiesis, wound healing, intestinal ischemia, day–night rhythm regulation, and angiogenesis in tumor models [6].

### 1.1. Synthesis and Degradation of Histamine

Histamine is formed by oxidative decarboxylation from the amino acid L-histidine with the enzyme histidine decarboxylase (HDC). Histamine is degraded as a result of the cyclopentyl action of histamine N-methyltransferase (HNMT) and by oxidative deamination of diaminoxidase (DAO). HNMT is mainly responsible for the degradation of intracellular histamine. The highest expression of HNMT occurs in the kidneys and liver as well as in the spleen, colon, prostate, ovary, cells in the spinal cord, bronchi, and trachea [7]. A small part of histamine is converted into N-methylhistamine by the action of HNMT. In its original form, approximately 2–3% of histamine is excreted [8,9]. DAO, which is a secreted protein, is responsible for the degradation of extracellular histamine. The greatest activity of DAO is recorded in the small intestine, colon, placenta, and kidneys. The vast majority of histamine is converted into imidazole acetic acid by DAO [9]. 

### 1.2. Sources of Histamine in the Body

The main cellular source of histamine are mast cells and basophils [10]. In the Golgi apparatus of the cell, the amino acid L-histidine is decarboxylated with the enzyme L-histidine decarboxylase, whose co-factor is pyridoxal phosphate (vitamin B6). The result of this reaction is the formation of histamine, which is later stored in the cytoplasmic granules along with other amines (e.g., serotonin), proteases, proteoglycans, cytokines/chemokines, and angiogenic factors and released after sensitization and degranulation of the cell [11,12]. The degranulation of mast cells and the release of histamine occur mainly as the result of binding a specific antigen to the FcRI receptor as well as in response to non-immune stimuli (e.g., neuropeptides, parts of the complement system, cytokines, platelet activation factor). IgE antibodies are mediators of mast cell degranulation during allergic diseases. The binding of IgE to its high-affinity IgE receptor on mast cell surfaces is called “sensitization” and precedes the development of clinical allergy. Histamine released from mast cells and basophils exerts its biological activities by activating four G protein-coupled receptors, namely H1R, H2R, H3R (expressed mainly in the brain), and H4R. While H1R and H2R activation mainly accounts for some mast cell and basophil-mediated allergic disorders, the selective expression of H4R on immune cells is uncovering new roles for histamine (possibly derived from mast cells and basophils) in allergic, inflammatory, and autoimmune disorders [12]. Histamine release also results from the action of a variety of chemical and physical factors such as extreme temperatures, trauma, vibrations, or alcohol [6]. Histamine can also be synthesized and released by other cell types (e.g., gastric enterochromaffin-like cells, histaminergic neurons, dendritic cells (DCs), T lymphocytes, platelets, etc. [10]).

It is estimated that about 5% of total histamine enters the body with food or is produced by intestinal microorganisms [13]. The most popular histamine-rich foods are fish and seafood, matured or fermented foods (e.g., cheese, alcohol, pickles, etc.), and some vegetables (e.g., spinach, eggplant, tomato, etc.). According to the law in the European Union, the permissible content of histamine in food is a maximum of 200 mg/kg in fresh fish and 400 mg/kg in seafood [14]. Histidine is produced mainly in autolytic or bacterial processes, therefore high concentrations of histamine are mainly found in microbial fermentation products [15]. The conditions for the formation of biogenic amines in food are the availability of free amino acids, the presence of decarboxylase-positive microorganisms as well as the conditions enabling the growth of bacteria and the activity of decarboxylase.

Microbiota are also an important source of histamine [16,17,18]. The production of histamine by bacteria in the human gut has been shown to influence the immune response. Therefore, elucidating the role of histamine as a metabolite of gut bacteria is an interesting area of research. Genes encoding HDC and synthesizing histamine have been demonstrated in many Gram-positive and Gram-negative bacteria. It was shown that in bacteria belonging to the genera Lactobacillus, Pediococcus, and Oenococcus, the presence of histidine is a factor inducing the expression of genes encoding HDC, while the presence of histamine caused the opposite effect [18]. Two HDC superfamilies have been described: Gram-negative bacteria have HDCs that require the presence of a coenzyme, which is pyridoxal phosphorus. In turn, for Gram-positive bacteria, covalently bonded pyruvate is used for catalysis [19]. The secretion of decarboxylase by bacteria is regulated by many factors (e.g., the presence of fermenting carbohydrates, oxygen, or chloride concentration). In an acidic environment, the expression of the activity of amino acid decarboxylases increases. This causes a local increase in pH around the bacteria and has a protective function [20]. The species of bacteria with the highest histidine decarboxylase activity are Morganella morganii, Eschericha coli, Hafnia alvei, Proteus vulgaris, Proteus milabilis, Enterobacter aerogenes, Raoultella planticola, Raoultella ornithinolytica, Citrobacter freundii, Pseudomonas fluorescens, and Photobacterium damselae [21]. Some bacteria have the ability to metabolize histamine. The aerobic growth of Pseudomonas putida U was demonstrated on a minimal medium, the only carbon source of which was histamine. In the six-stage catabolic process, histamine is converted into aspartic acid, which is then converted into fumaric acid. It has been shown that 11 proteins (HinABCDFLHGIJK) are necessary for the metabolism of histamine in P. Putida U. Genome studies indicate that Hin genes are present in strains of the genus Pseudomonas, but have not been shown to be present in previously sequenced Gram-positive bacteria [22]. Depending on the type of activated histamine receptor, it exerts either a pro-inflammatory or anti-inflammatory effect. Histamine derived from Lactobacillus reuteri via histamine receptor 2 inhibited the production of tumor necrosis factor-α (TNF-α) (induced by toll-like receptor) by human monocytoid cells [23]. In an experimental mice model, the immunomodulatory effect of histamine secreted by Lactobacillus rhamnosus was demonstrated. In mice without the deficiency of H2R, administration of this bacterial strain induced an anti-inflammatory effect (decreased secretion of interleukins, TNF-α) [24]. Some reports indicate that the amount of histamine secreted may determine its pathophysiological effects. These assumptions were confirmed by the study with Lactobacillus saerimneri, synthesizing almost 100 times more histamine compared to L. rhamnosus. Consequently, apart from various immunological effects, a decrease in the body weight of the animals and deterioration of the general condition were also observed [25]. The effect of bacterial secretion of histamine on intestinal diseases and digestive disorders has been reported. Therefore, it is important to deepen the knowledge of the factors influencing the synthesis, release, and metabolism of histamine by bacterial strains that make up the intestinal microbiome. 

External factors that reduce the microbial diversity may cause differences in the composition of the intestinal microbiota, which may result in a state of dysbiosis. The exact mechanisms leading to dysbiosis remain unclear. The combination of physiological changes and the action of stress factors should be taken into account. Research indicates a relevant relationship between intestinal dysbiosis and the occurrence of intestinal diseases (e.g., inflammatory bowel diseases, histamine intolerance, irritable bowel syndrome) [26].

## 2. Histamine Scheme of Action through Histamine Receptors

**Figure 1:**
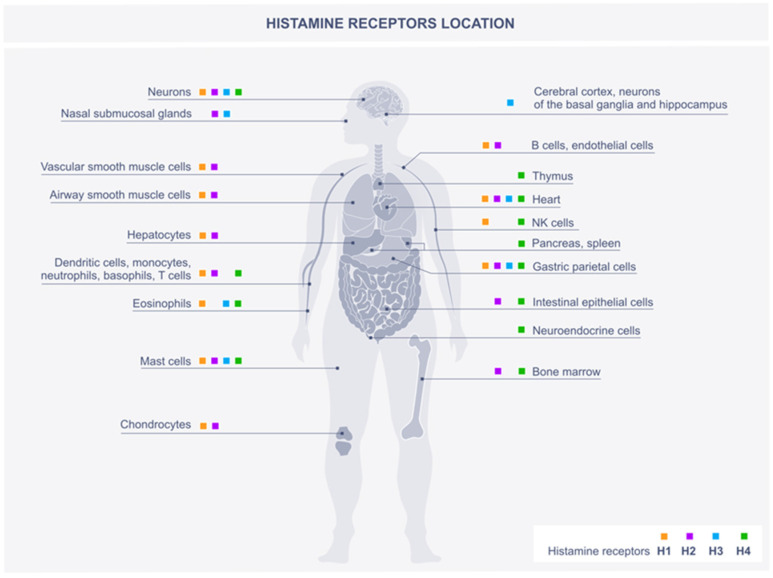
The location of histamine receptors in the human body.

The effects of histamine are due to the activation of four histamine receptor (HR) subtypes—H1R, H2R, H3R, and H4R [20,27]. Histamine receptors belong to the rhodopsin-like family of G-protein coupled receptors and are differentially expressed in numerous cell types. The tissue preference of histamine receptors are shown in Figure 1. They differ in their signaling mechanisms [28], but simultaneous activation of more than one receptor on a specific cell can lead to altered effects [29].

### 2.1. H1R

H1 receptor activation occurs through the Gαq/11 protein, which causes the activation of phospholipase C and an increase in Ca^2+^ levels [30]. The effect of H1R activation is the contraction of airway smooth muscles, an increase in vascular permeability as well as the induction of the production of prostacyclin and platelet activating factor [31]. H1R is present in many types of cells (e.g., neurons, airway smooth muscle cells, chondrocytes, hepatocytes, endothelial cells, dendritic cells, monocytes, neutrophils, T cells, and B cells [32]). The H1 receptor is the main receptor involved in the development of allergic reactions. Allergic symptoms such as redness, itching, and swelling are related to IgE-mediated activation of mucosal mast cells. Activation of these cells results in the release of histamine and other mediators from their granularity [11]. Activation of H1R in murine models induces an increase in IFN (interferon) production, which is associated with the proliferation of type 1 T helper cells and induces a proinflammatory effect [33]. It has been shown that the expression of pruritic factors (e.g., nerve growth factor, semaphorin 3A) is regulated by histamine H1R. In murine models and patients with atopic dermatitis, the use of an H1R antagonist resulted in a reduction in IL-31 (interleukin-31) levels, which is associated with the onset of pruritus [34].

### 2.2. H2R

H2R expression has been observed in the digestive system (e.g., gastric parietal cells, enterocytes), smooth muscle cells, cardiomyocytes, dendritic cells (DC), and also in T and B cells. H2R receptors are postsynaptic, transmitting signals mainly via cyclic adenosine monophosphorane (cAMP) and coupling with Gαs [35]. H2R stimulation causes the external secretion of hydrochloric acid, relaxation of smooth muscle cells, and tachycardia. H2R stimulation also causes anti-inflammatory effects by inhibiting the production of IL-12, IFN-γ, TNF-α cytokines by monocytes or macrophages and mast cells, reducing the proliferation of T-helper 1 and the production of antibodies [36]. During the binding of histamine to the H2R, there is an increase in IL-10 secretion and a decrease in IL-12 levels. Consequently, DC with histamine maturation polarized naive CD4+ T cells toward the Th2 phenotype. This study suggests that Th2 cells stimulate IgE production, which may induce increased secretion of histamine by mast cells. This effect may constitute a positive feedback loop and contribute to the aggravation of atopic diseases [37]. Histamine (endogenous and exogenous) significantly changes the innate immune response to microorganisms through H2R [24]. Knockdown of H2R−/− mice caused disorders of the immune system as well as gastric defects (decreased gastric acid secretion). Cognitive decline and abnormal nociception have also been observed [38,39,40].

### 2.3. H3R

The expression of H3 receptors is observed in cells of the nervous system, especially in the cerebral cortex, neurons of the basal ganglia, and the hippocampus. H3Rs are located in the presynaptic region of histamine-containing neurons. Their function is to regulate the synthesis and release of histamine as well as other neurotransmitters (e.g., dopamine, norepinephrine, gamma-aminobutyric acid, acetylcholine, and serotonin) [41]. Changes in the expression and activation of H3 receptors play an important role in sleep–wake cycle disorders, attention deficit hyperactivity disorder, epilepsy, and cognitive disorders as well as in the development of inflammation [42]. In studies in mouse models, the induction of acetylcholine release by H3R antagonists has also been shown to increase insulin secretion as well as significantly reduce the total body weight and triglyceride levels in obese mice. This effect may be related to inducing a feeling of fullness in the hypothalamus. The hypoglycemic effect of the H3R antagonist is comparable to that of metformin [43]. H3R stimulation increases pro-inflammatory activity as well as the ability of immune cells to present the antigen. Potentially, the use of histamine H3R antagonists could be used in preventing or inhibiting the development of inflammatory diseases (e.g., in the respiratory system) [44].

### 2.4. H4R

H4 receptors were discovered the most recently and their role is not yet fully understood. H4Rs are present mainly in immune cells (eosinophils, basophils, mast cells, natural killer (NK) cells, DC cells, monocytes, and T cells) and also in the spleen, thymus, bone marrow, intestinal epithelium, and neuroendocrine cells. They are also found in the bile and pancreatic ducts [45]. In contrast to other types of histamine receptors, the H4 receptor is not particularly expressed in the central and peripheral nervous system [46]. H4 receptors are coupled to Gi proteins and their downstream pathways are believed to be similar to those described for H3Rs. H4R activation is an important factor modeling chemotaxis as well as other cell functions. As a result of H4R-mediated activation of mast cells, pro-inflammatory cytokines and chemokines IL-6, TNF-α, TGF-β1 (tumor growth factor-β1), RANTES, IL-8, MIP-1α, and MCP-1 are expressed [47]. The available studies suggest that H4Rs, through interactions with Gα/io proteins, contribute to the development of inflammatory reactions and hypersensitivity [12]. Activation of H4Rs has been shown to lead to pruritus. The use of H4 antagonists had an antipruritic effect, and the simultaneous blockade of H1 and H4Rs enhanced this effect. Research suggests that H4R, by activating Th2 cells and producing IL-31, may trigger the development of allergic dermatitis [48]. Activation of H4R and H3R increases the effect of acetylcholine on the peristaltic movement of the intestines [49]. H4 receptors are also involved in peptic ulcer formation and carcinogenesis [50].

## 3. Histamine in the Intestines

Histamine is well-recognized for its effects in the immediate type hypersensitivity response (type I of hypersensitivity reactions by Gel–Coombs classification). The pathological effect of increased levels of histamine in the gut is less understood. However, as described further in this review article, the increased levels of histamine alter the host immune interactions with microbiota and lead to a breakdown in homeostasis, causing the development of many gut diseases that are difficult to cope with. Histamine levels in the gut can be influenced by host allergic and inflammatory responses, somehow altering the activity of enzymes that degrade or synthesize histamine as well as its dietary intake in addition to the host microbiota production. Furthermore, the amount of endogenous levels of histamine can be enhanced upon the stimulation of histamine-producing immune cells. All of this can influence gut homeostasis, lead to histamine accumulation, and breakdown to a specific disorder. The changed interactions with histamine receptors caused by their different synthesis can lead to further implications. Agonists or antagonists of histamine receptors or both added simultaneously will further modify this already complicated scenario. On top of this are environmental factors and genetic predisposition. This makes it a very complicated problem to deal with from the therapeutic point of view. Histamine may also negatively or positively influence the parasitic and bacterial infections [51,52]. All histamine receptors except H3R are also expressed throughout the intestinal tract in humans [53]. From a quantitative point of view, H4R expression is significantly less abundant in comparison to H1R and H2R, at least on the mRNA level [45,54,55]. 

## 4. Role of Histamine in Intestine Disorders

**Figure 2:**
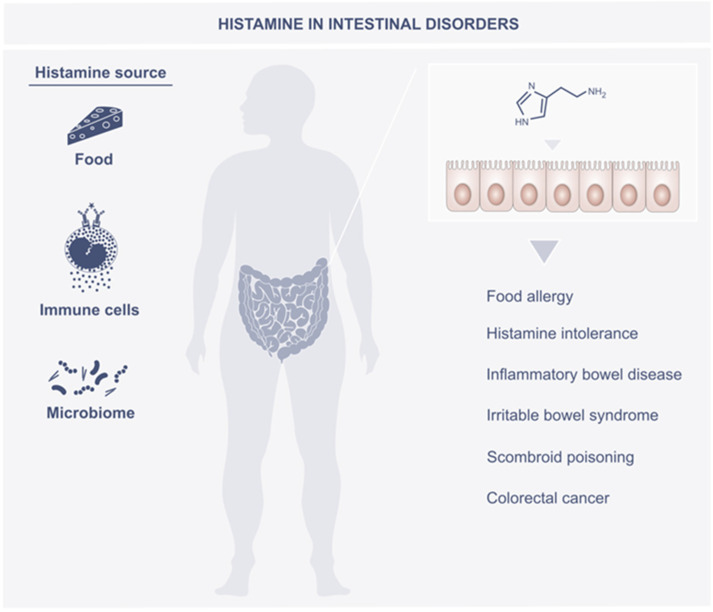
Histamine in intestine disorders.

The role of histamine in intestinal disorders is schematically shown in Figure 2.

### 4.1. Food Allergy

The digestive tract is a place that comes into contact with a large number of different molecules that are potential allergens. The characteristic symptoms of food allergies are manifested in the respiratory, digestive, cardiovascular, and skin systems. IgE-dependent food allergies develop as a result of disorders of the immune system, leading to a loss of tolerance. This leads to the recognition of mild food antigens as pathogens. Taking into account the described immunoregulatory functions of histamine, it is presumed that it may alter the immune response of the intestines to food antigens. Research results indicate the participation of histamine receptors in the development of food allergies [20]. It has been shown that the use of H2R antagonists in humans increased the production of IgE against food antigens [56]. The pathophysiology of IgE-dependent food allergy is related to the activation of the immune system. In response to a stimulus from Th2 cells, IgE binds to Fcε receptors on effector cells (mast cells and basophils). As a result of the activation of effector cells, histamine is released as well as other mediators. As a result of the IgE-dependent reaction, clinical symptoms are rapidly manifested [57]. In a food-allergic subject, enhanced secretion of histamine and increased numbers of mast cell were well-demonstrated [58,59,60]. For example, histamine release from basophils was positively correlated with the skin prick test, and food challenge. The anti-IgE-mediated mast cell histamine release in food-allergic patients was increased compared to the non-allergic [61]. Additionally, the incubation of biopsies from food-allergic patients with anti-IgE (human) antibodies or allergens induced a ninefold increase in histamine release. Further stimulation of biopsis ex vivo with histamine induced a concentration-dependent NO response only in the food allergic patients [62]. Food allergy can manifest as mild and severe symptoms. The most severe, potentially life-threatening manifestation is anaphylaxis. Strict avoidance of food allergens is a long-term strategy for managing IgE-mediated food allergies. Food allergy is diagnosed on the basis of clinical symptoms, skin prick tests, and the presence of specific IgE in the serum. However, the gold standard in diagnosing food allergy is to complete the double-blind placebo controlled food challenge (DBPCFC) [63]. In this method, neither the patient nor the doctor knows what food allergen is administered. The patient is exposed to gradually increasing doses of the suspected food, hidden in a matrix. The DBPCFC can be performed in a 1- or a 2-day approach. During a 1-day approach, placebo doses containing an identical matrix are randomly interspersed. During a 2-day approach, one day consists of verum doses, and one day of the placebo. The order of the verum and placebo days are random. The DBPCFC is performed in a hospital setting, with a trained nurse and full emergency medication readily available. The challenge is discontinued when objective symptoms occur, or when consistent subjective symptoms occur on at least three subsequent doses. Due to the increasing incidence of food allergies, numerous studies have been conducted to develop new therapeutic and preventive strategies. There are also many studies on various stages of the pathogenesis of food allergies such as the influence on the Th2 pathways, blocking IgE, suppression of effector cells, and microbial therapeutics [64]. Long-term immune tolerance should be the most desirable effect in the treatment of food allergy [65]. Oral immunotherapy (OIT) is one of the developing treatments of food allergies. It consists in administering allergens to patients in doses increased every 2–4 weeks, until the maximum maintenance dose is reached. The result of this procedure is the development of tolerance to food. This method has been used in food allergies to milk, eggs, wheat, peanuts, nuts, and shellfish [66]. The FDA has approved oral immunotherapy for peanut allergy [67]. Epidermal and sublingual immunotherapy are currently under investigation. Clinical trials have also been conducted on epidermal immunotherapy in the case of allergies to milk and eggs.

### 4.2. Histamine Intolerance

Histamine intolerance (HIT) is a condition in which, due to the reduced ability to break down histamine, it accumulates [68]. In other words, there is no balance between accumulated histamine and the capacity for its degradation. In healthy patients, the intestinal epithelial cells have an enzyme barrier created by DAO and HMNT. This barrier prevents excessive resorption of exogenous histamine in the bloodstream. If these enzymes are inhibited or reduced, symptoms of histamine intolerance may occur after consuming even a small amount of histamine [69]. An underlying cause of histamine intolerance is diamine oxidase (DAO) deficiency, which leads to defective homeostasis and a higher systemic absorption of histamine. Impaired DAO activity may have a genetic, pharmacological, or pathological origin. The decrease in DAO activity may be caused by damage to enterocytes in the course of gastrointestinal diseases (e.g., inflammatory bowel diseases, infections). Other biogenic amines, drugs, and alcohol may inhibit the action of DAO. The microbiome also influences the development of histamine intolerance. A recent proposal also suggests that HIT can arise from an alteration in the gut microbiota [70]. A greater abundance of histamine-secreting bacteria in the gut could lead to the development of histamine intolerance. A greater number of the Bifidobacteriaceu family in healthy people has been shown. Higher numbers of the genus Proteobacteria were observed in people with decreased serum DAO activity [71]. 

Dysbiosis of the gut microbiota was observed in the histamine intolerance group who, in comparison with the healthy individuals, had a significantly lower proportion of *Prevotellaceae*, *Ruminococcus*, *Faecalibacterium*, and *Faecablibacterium prausnitzii*, which are bacteria related to gut health. They also had a significantly higher abundance of histamine-secreting bacteria including the genera *Staphylococcus* and *Proteus*, several unidentified genera belonging to the family *Enterobacteriaceae*, and the species *Clostridium perfringens* and *Enterococcus faecalis*. A greater abundance of histaminogenic bacteria would favor the accumulation of high levels of histamine in the gut, its subsequent absorption in plasma, and the appearance of adverse effects, even in individuals without DAO deficiency, as the study by Sánchez-Pérez and co-authors showed [72]. Both genetic and environmental factors contribute to the development of HIT. Single nucleotide polymorphism of the single nucleotide polymorphism in the DAO gene results in altered production of a protein with lower enzymatic activity [8]. Increasing the amount of histamine metabolites leads to the inhibition of the second histamine metabolizing enzyme—HNMT [7]. There have also been speculation that histamine intolerance may be a metabolic disease [73]. Aside from problems with histamine digestion, a possible cause of HIT is endogenous histamine overproduction or increased exogenous ingestion of histidine or histamine from food. A plasma histamine concentration of 0.3 to 1.0 ng/mL is considered normal [74]. Common symptoms of HIT appear as a result of an increase in the levels of histamine in the body. When exposed to large amounts of histamine, even in healthy people, symptoms such as severe headache and hot flushes may occur. This effect is known as scromboid poisoning. Secondary HIT symptoms are related to the synthesis and release of catecholamines, which is caused by the increased concentration of histamine. This can cause a paradoxical increase in blood pressure, tachycardia, arrhythmias, nervousness, and sleep disturbance [6]. Symptoms of histamine intolerance are presented in Table 1.

Clinical diagnosis of HIT remains a challenge, as standardized diagnostic tests are lacking. The diagnosis of histamine intolerance can be made only after excluding other causes that may produce similar symptoms. IgE-mediated food allergy, mastocytosis, and the action of drugs that may interfere with the metabolism and distribution of histamine should be ruled out. Diagnosis usually requires the presence of at least two clinical symptoms in less than four hours after food intake and their improvement or remission after a low-histamine diet. Complementary tests such as the determination of DAO activity in blood samples or intestinal biopsy and the identification of genetic and metabolic markers are also available [8,75]. The gold standard of treatment is a low-histamine diet. A good response to 4–8 weeks of such a diet is considered to confirm the diagnosis of histamine intolerance. DAO supplementation is also recommended as a complementary treatment in people with intestinal DAO deficiency [75,76]. In severe conditions where a low-histamine diet is insufficient, H1R antihistamines can be used for a short time. Some studies have shown that supplementation with DAO enzyme cofactors such as vitamin C, copper, and vitamin B6 may be an adjunctive therapy [77]. An interesting field of research seems to be supplementation with probiotic microorganisms. Such an approach could lead to a reduction in the production of the bacterial L-histidine decarboxylase enzyme as well as to the simultaneous degradation of histamine (or other biogenic amines). There are no studies assessing the effectiveness of supplementation with probiotic microorganisms. However, based on the available information, it can be assumed that members of the genus Bifidobacterium may be considered as candidates for adequate supplementation [71,78].

### 4.3. Inflammatory Bowel Disease

Inflammatory bowel diseases (IBD) are idiopathic, chronic-recurring diseases of the gut. Their two main manifestations, ulcerative colitis (UC) and Lesniowski–Crohn’s disease (CD), differ in their clinical, endoscopic, and histologic appearance. In CD, the inflammation appears in diffuse lesions that can be found all over the digestive tract and deeply penetrates the intestinal wall, possibly affecting all layers. In contrast, inflammatory lesions in UC start in the rectum, proceed upward but do not exceed the colon, and remain superficial at the mucosa. Lesniowski–Crohn disease leads to transmural inflammation throughout the whole gastrointestinal tract but is characterized by a discontinuous pattern. In contrast to ulcerative colitis where inflammation is superficial, ulcerations are limited mainly to the colon mucosa. IBD decreases the quality of the patients’ lives and not treated, can be life threatening. Both manifestations present similar symptoms (e.g., mucosal lesions, ulcers, edema, diarrhea, bloody stool, abdominal pain). Studying the available knowledge about the mechanisms of these diseases, it is easy to conclude that both disease development is a result of complex interactions between the host immune system, enteric microbiota, and environmental factors in genetically susceptible patients. Mucosal histamine levels (not plasma levels) are increased in patients with IBD. Increased levels of N-methylhistamine that correlated with disease activity were found in the patients’ urine [59,79,80]. Mast cells originating from the resected colon of active Lesniowski–Crohn’s disease or ulcerative colitis were able to release more histamine than those from the normal colon when being stimulated with an antigen, colon-derived murine epithelial cell-associated compounds [14]. Similarly, cultured colorectal endoscopic samples from patients with IBD secreted more histamine toward substance P alone or substance P with anti-IgE than the samples from normal control subjects under the same stimulation [15]. The histamine signaling pathway is disrupted in both patients with Lesniowski–Crohn disease and ulcerative colitis, as shown in the detailed analysis conducted by Smolinska and co-authors [81]. Histamine receptor expression and functional activity is altered in IBD patients. In addition blockage of H2R resulted in more severe inflammatory disease in the murine T-cell transfer colitis model. Histamine mostly suppresses IFN-γ and TNF-α secretion, and the gene expression of these cytokines correlates positively with H4R and H2R expression accordingly in patients with ulcerative colitis. HNMT gene expression is reduced in inflamed mucosa, and DAO polymorphisms have been associated with an increased risk of IBD [81,82,83]. The use of the H2R antagonist (but not proton pump inhibitors) increases the risk of hospitalization or surgery in Lesniowski–Crohn disease patients [42]. Transfer of T cells, which lack H2R or inhibit H2R using femotidine (H2R antagonist), accelerates weight loss and increases the disease severity in a mouse colitis model. Patients with IBD are treated with anti-inflammatory drugs, steroids, antibiotics, aminosalicylates, or welcome biological therapy with the use of infliximab (anti-TNF-α). In many cases, the only option to obtain a state of remission is radical surgery, where the inflamed areas are cut out. Potentially, the use of an antagonist for H1R and H4R with the simultaneous use of the H2R agonist can be beneficial for patients with IBD [81].

### 4.4. Irritable Bowel Syndrome

Functional dyspepsia (FD), irritable bowel syndrome (IBS), and small intestinal bacterial overgrowth (SIBO) are commonly reported, but solely as symptom-oriented conditions. These clinical syndromes continue to be imprecise and were therefore re-named to “IBS-like” disorders [84]. There is a lack of specificity of symptoms. Abdominal pain, diarrhea, nausea, vomiting, etc. are general symptoms linked with gastrointestinal disorders. IBS is a chronic condition linked to abdominal discomfort or pain where the food eaten is a trigger of more severe symptoms. Some evidence has shown that the gastrointestinal microbiota is altered and perhaps this disrupted mucosal immune response plays a significant role [85,86]. In one study, more than half of the patients experienced gastrointestinal symptoms from histamine-releasing food items and foods rich in biogenic amine [87]. The level of endogenous histamine definitely correlates with the severity of symptoms in IBS patients. Activated mast cells produced higher amounts of histamine, which correlated with abdominal pain in IBS patients [88]. Mucosal biopsy supernatants from IBS patients contained higher levels of histamine compared to supernatants delivered the same way from healthy subjects [89,90]. Histamine levels and the abundance of HDC genes were determined in both healthy and IBS patients using metabolomics and metagenomics data from the integrative Human Microbiome Project. These analyses revealed that IBS patients presented higher levels of histamine and bacterial HDC genes [91]. Subsequent studies have also shown that supernatants from colonic samples of IBS patients contained increased histamine levels, and the expression levels of histamine receptors H1R and H2R were upregulated in IBS patients [92]. The authors thus hypothesized that a dysbiosis with increased histamine-secreting or HDC-containing bacteria was potentially associated with the development and aggravation of IBS [93]. Administration of specific microbes has therapeutic effects, which can also be an argument for microbiota changes as a cause of disease [94,95,96]. There is no specific treatment for IBS. Drugs that decrease inflammation are in use.

### 4.5. Scombroid Poisoning

Scombroid poisoning or histamine fish poisoning results from mishandled fish [97]. The symptoms are variable and can include oral numbness, headache, dizziness, palpitations, low blood pressure, difficulties in swallowing, week or rapid pulse, hives, rush, flushing, swelling of face, vomiting, nausea, and diarrhea. Histamine is generated in fish tissue by bacterial conversion of free histidine by a wide range of bacterial species: Morganella morganii, Enterobacter aerogenes, Raoultella planticola, Raoultella ornithinolytica, and Photobacterium damselae. Aside from high levels of histamine, its toxicity can be enhanced by inhibitors of enzymes that degrade histamine. DAO and HNMT inhibitors were also present in the ingested fish [98]. In addition, toxins that induce mast cell degranulation are found in spoiled fish, leading to further increases of histamine in the host body [99]. Furthermore, some substances that have the potential to be histamine receptor blockers were also found in the fish. The symptoms of scombroid disease are usually rapid and do not last longer than 24 h. Treatment includes the administration of antihistamines.

### 4.6. Colorectal Cancer

Colorectal cancer (CRC) is the third most common cancer and the third leading cause of cancer-related mortality [100]. Patients with inflammatory bowel disease have an increased lifetime risk of CRC compared with the general population [101,102]. This risk can be reduced by the treatment of colitis with the suppression of intestinal inflammation [103]. The role of the intestinal microbiome in colon cancer development has been investigated [104,105,106,107]. Specific gut microbes and their metabolites may contribute to the cause of CRC [108,109,110]. Histidine decarboxylase deficiency has been shown to promote inflammation-associated colorectal cancer by the accumulation of CD11b^+^Gr-1^+^ immature myeloid cells, indicating a potential antitumorigenic effect of histamine. Several probiotic strains including *Bifidobacterium* *longum* [111], *Lactobacillus acidophilus* NCFM [112], and *Lactobacillus rhamnosus* GG [113] have shown beneficial effects in different murine models of colon cancer. This histamine-producing probiotic decreased the number and size of colon tumors and the colonic uptake of [^18^F]-fluorodeoxyglucose by positron emission tomography in *HDC*^−/−^ mice. Administration of *L. reuteri* suppressed keratinocyte chemoattractant (*KC*), *Il22*, *Il6*, *Tnf*, and *IL1α* gene expression in the colonic mucosa and reduced the amounts of proinflammatory, cancer-associated cytokines, keratinocyte chemoattractant, IL-22, and IL-6, in plasma. Histamine-generating *L. reuteri* also decreased the relative numbers of splenic CD11b^+^Gr-1^+^ immature myeloid cells. Furthermore, an isogenic HDC-deficient *L. reuteri* mutant that was unable to generate histamine did not suppress carcinogenesis, indicating a significant role of the cometabolite, histamine, in the suppression of chronic intestinal inflammation and colorectal tumorigenesis. In the colonic mucosa of CRC patients, HDC activity as well as histamine content were increased in comparison to the normal samples [114,115]. However, in mice with experimentally induced CRC, the deletion of HDC resulted in enhanced tumorigenesis compared to the wild type mice, pointing toward an anti-carcinogenic effect of histamine, which is supported by the finding that gut microbiota-derived histamine suppresses colorectal tumorigenesis [116]. On one hand, histamine promotes the underlying inflammatory process, leading to tumor initiation. On the other hand, histamine in the tumor’s tissue may affect the differentiation of immature myeloid cells toward neutrophils and myeloid-derived suppressor cells, both resulting in tumor regression [117,118]. While the effect of histamine on the differentiation toward neutrophils is a direct one, the differentiation of myeloid-derived suppressor cells is affected by IL-17, which is produced by tumor-associated mast cells upon histamine stimulation. This anti-cancer effect of histamine is supported by similar findings obtained in models of esophageal squamous carcinoma [119]. Interestingly, mast cells have been found to be abundant in colon carcinoma and to promote carcinogenesis in chemically-induced CRC in mice [120], and are associated with a poor prognosis in human CRC patients [121]. In analogy to the pro-inflammatory effect of histamine via the H4R, the absence of H4R expression also leads to a reduction in chemically-induced carcinogenesis in mice [122]. However, some indications arise from the observation that the expression of H4R decreases in gastric carcinoma during progression, accompanied by the attenuated histamine-induced suppression of proliferation [119,123]. 

## 5. Conclusions

Histamine is a mediator that is mainly recognized due to its role in inducing allergic symptoms, but it is also involved in non-allergic inflammatory reactions. The role of histamine present within the gastrointestinal mucosa is of special interest. Its potential seems to be underestimated. In some concentration ranges, histamine plays a protective role and is pivotal to maintain the healthy status. However, at higher concentrations histamine contributes to the pathophysiology of mucosal inflammatory disorders. The overlap of various mechanisms complicates the understanding of its role in disease and the possible design of diagnostics and curative modalities based on them. The application of various medications that utilize mechanisms interfering with histamine signals could be beneficial for patients. Enhancement of H2R expression and/or its intracellular signals with simultaneous decrease H1R or/and H4R activity is a plausible approach to improve mucosal immunity including a protective umbrella in both allergy and autoimmunity. The use of gut microbiota with the potential to release histamine offers a novel therapeutic perspective. 

## Figures and Tables

**Table 1:** Symptoms of histamine intolerance from a specific organ.

	Symptoms of Histamine Intolerance
Respiratory system	rhinorrhea, rhinitis, nasal congestion, dyspnea, sneezing
Cardiovascular system	tachycardia, hypotonia, collapse
Gastrointestinal system	bloating, flatulence, postprandial fullness, diarrhea, abdominal pain, constipation, nausea, vomiting
Reproductive system	menstrual cramps, dysmenorrhea
Skin	pruritus, flushing, urticarial, dermatitis, swelling
Nervous system	headache/migraine, dizziness, chronic inappropriate fatigue, nervousness, sleep disturbances (insomnia), anxiety, panic disorder, depression

## References

[1] Barger G., Dale H.H. (1910). Chemical structure and sympathomimetic action of amines. J. Physiol..

[2] Dale H.H., Laidlaw P.P. (1910). The physiological action of β-iminazolylethylamine. J. Physiol..

[3] Lindskog M. (2017). Histamine Receptors in the Cross-Talk between Periphery and Brain. Int. J. Neuropsychopharmacol..

[4] Hungerford J.M. (2010). Scombroid poisoning: A review. Toxicon.

[5] Obara I., Telezhkin V., AlRashdi I., Chazot P. (2020). Histamine, histamine receptors, and neuropathic pain relief. Br. J. Pharmacol..

[6] Kovacova-Hanuskova E., Buday T., Gavliakova S., Plevkova J. (2015). Histamine, histamine intoxication and intolerance. Allergol. Immunopathol..

[7] Maintz L., Novak N. (2007). Histamine and histamine intolerance. Am. J. Clin. Nutr..

[8] Comas-Basté O., Sánchez-Pérez S., Veciana-Nogués M.T., Latorre-Moratalla M., Vidal-Carou M.D.C. (2020). Histamine Intolerance: The Current State of the Art. Biomolecules.

[9] Schwelberger H.G., Hittmair A., Kohlwein S.D. (1998). Analysis of tissue and subcellular localization of mammalian diamine oxidase by confocal laser scanning fluorescence microscopy. Inflamm. Res..

[10] Panula P., Chazot P.L., Cowart M., Gutzmer R., Leurs R., Liu W.L.S., Stark H., Thurmond R.L., Haas H.L. (2015). International Union of Basic and Clinical Pharmacology. XCVIII. Histamine Receptors. Pharmacol. Rev..

[11] Zhang Z., Kurashima Y. (2021). Two Sides of the Coin: Mast Cells as a Key Regulator of Allergy and Acute/Chronic Inflammation. Cells.

[12] Borriello F., Iannone R., Marone G. (2017). Histamine Release from Mast Cells and Basophils. Histamine Histamine Recept. Health Dis..

[13] Landete J.M., Pardo I., Ferrer S. (2006). Histamine, Histidine, and Growth-Phase Mediated Regulation of the Histidine Decarboxylase Gene in Lactic Acid Bacteria Isolated from Wine. FEMS Microbiol. Lett..

[14] Visciano P., Schirone M., Tofalo R., Suzzi G. (2014). Histamine poisoning and control measures in fish and fishery products. Front. Microbiol..

[15] Zhao Y., Zhang X., Jin H., Chen L., Ji J., Zhang Z. (2022). Histamine Intolerance-A Kind of Pseudoallergic Reaction. Biomolecules.

[16] Barcik W., Pugin B., Westermann P., Perez N.R., Ferstl R., Wawrzyniak M., Smolinska S., Jutel M., Hessel E.M., Michalovich D. (2016). Histamine-secreting microbes are increased in the gut of adult asthma patients. J. Allergy Clin. Immunol..

[17] Smolinska S., O’Mahony L. (2016). Microbiome—Host Immune System Interactions. Semin. Liver Dis..

[18] Krell T., Gavira J., Velando F., Fernández M., Roca A., Monteagudo-Cascales E., Matilla M. (2021). Histamine: A Bacterial Signal Molecule. Int. J. Mol. Sci..

[19] Landete J.M., De las Rivas B., Marcobal A., Muñoz R. (2008). Updated molecular knowledge about histamine biosynthesis by bacteria. Crit. Rev. Food Sci. Nutr..

[20] Smolinska S., Jutel M., Crameri R., O’Mahony L. (2014). Histamine and gut mucosal immune regulation. Allergy.

[21] Barcik W., Wawrzyniak M., Akdis C.A., O’Mahony L. (2017). Immune regulation by histamine and histamine-secreting bacteria. Curr. Opin. Immunol..

[22] De la Torre M., Gomez-Botran J.L., Olivera E.R., Bermejo F., Rodríguez-Morán J., Luengo J.M. (2018). Histamine catabolism in Pseudomonas putida U: Identification of the genes, catabolic enzymes and regulators. Environ. Microbiol..

[23] Thomas C.M., Hong T., Van Pijkeren J.P., Hemarajata P., Trinh D.V., Hu W., Britton R.A., Kalkum M., Versalovic J. (2012). Histamine derived from probiotic lactobacillus reuteri suppresses TNF via modulation of PKA and ERK signaling. PLoS ONE.

[24] Frei R., Ferstl R., Konieczna P., Ziegler M., Simon T., Rugeles T.M., Mailand S., Watanabe T., Lauener R., Akdis C.A. (2013). Histamine receptor 2 modifies dendritic cell responses to microbial ligands. J. Allergy Clin. Immunol..

[25] Ferstl R., Frei R., Schiavi E., Konieczna P., Barcik W., Ziegler M., Lauener R.P., Chassard C., Lacroix C., Akdis C.A. (2014). Histamine receptor 2 is a key influence in immune responses to intestinal histamine-secreting microbes. J. Allergy Clin. Immunol..

[26] Weiss G.A., Hennet T. (2017). Mechanisms and consequences of intestinal dysbiosis. Cell. Mol. Life Sci..

[27] Carthy E., Ellender T. (2021). Histamine, Neuroinflammation and Neurodevelopment: A Review. Front. Neurosci..

[28] Shulpekova Y.O., Nechaev V.M., Popova I.R., Deeva T.A., Kopylov A.T., Malsagova K.A., Kaysheva A.L., Ivashkin V.T. (2021). Food Intolerance: The Role of Histamine. Nutrients.

[29] Smolinska S., Groeger D. (2017). Biology of the Microbiome 1: Interactions with the Host Immune Response. Gastroenterol. Clin. N. Am..

[30] Xia R., Na Wang N., Xu Z., Lu Y., Song J., Zhang A., Guo C., He Y. (2021). Cryo-EM structure of the human histamine H1 receptor/Gq complex. Nat. Commun..

[31] Simons F.E.R. (2004). Advances in H_1_-Antihistamines. New Engl. J. Med..

[32] Togias A. (2003). H1-receptors: Localization and role in airway physiology and in immune functions. J. Allergy Clin. Immunol..

[33] Jutel M., Klunker S., Akdis M., Małolepszy J., Thomet O.A., Żak-Nejmark T., Blaser K., Akdis C. (2001). Histamine Upregulates Th1 and Downregulates Th2 Responses due to Different Patterns of Surface Histamine 1 and 2 Receptor Expression. Int. Arch. Allergy Immunol..

[34] Ohsawa Y., Hirasawa N. (2014). The Role of Histamine H1 and H4 Receptors in Atopic Dermatitis: From Basic Research to Clinical Study. Allergol. Int..

[35] Park P., Sanderson T.M., Amici M., Choi S.-L., Bortolotto Z.A., Zhuo M., Kaang B.-K., Collingridge G.L. (2016). Calcium-Permeable AMPA Receptors Mediate the Induction of the Protein Kinase A-Dependent Component of Long-Term Potentiation in the Hippocampus. J. Neurosci..

[36] Shi Z., Fultz R., Engevik M.A., Gao C., Hall A., Major A., Mori-Akiyama Y., Versalovic J. (2019). Distinct roles of histamine H1- and H2-receptor signaling pathways in inflammation-associated colonic tumorigenesis. Am. J. Physiol. Gastrointest. Liver Physiol..

[37] Mazzoni A., Young H.A., Spitzer J.H., Visintin A., Segal D.M. (2001). Histamine regulates cytokine production in maturing dendritic cells, resulting in altered T cell polarization. J. Clin. Investig..

[38] Teuscher C., Poynter M., Offner H., Zamora A., Watanabe T., Fillmore P.D., Zachary J.F., Blankenhorn E.P. (2004). Attenuation of Th1 Effector Cell Responses and Susceptibility to Experimental Allergic Encephalomyelitis in Histamine H2 Receptor Knockout Mice Is Due to Dysregulation of Cytokine Production by Antigen-Presenting Cells. Am. J. Pathol..

[39] Dai H., Kaneko K., Kato H., Fujii S., Jing Y., Xu A., Sakurai E., Kato M., Okamura N., Kuramasu A. (2007). Selective cognitive dysfunction in mice lacking histamine H1 and H2 receptors. Neurosci. Res..

[40] Mobarakeh J.I., Takahashi K., Sakurada S., Kuramasu A., Yanai K. (2006). Enhanced antinociceptive effects of morphine in histamine H2 receptor gene knockout mice. Neuropharmacology.

[41] Nieto-Alamilla G., Márquez-Gómez R., García-Gálvez A.-M., Morales-Figueroa G.-E., Arias-Montaño J.-A. (2016). The Histamine H_3_ Receptor: Structure, Pharmacology, and Function. Mol. Pharmacol..

[42] Singh M., Jadhav H.R. (2013). Histamine H3 Receptor Function and Ligands: Recent Developments. Mini-Rev. Med. Chem..

[43] Kotańska M., Kuder K.J., Szczepańska K., Sapa J., Kieć-Kononowicz K. (2018). The histamine H3 receptor inverse agonist pitolisant reduces body weight in obese mice. Naunyn-Schmiedeberg’s Arch. Pharmacol..

[44] Abdulrazzaq Y.M., Bastaki S.M., Adeghate E. (2022). Histamine H3 receptor antagonists—Roles in neurological and endocrine diseases and diabetes mellitus. Biomed. Pharmacother..

[45] Sander L.E., Lorentz A., Sellge G., Coëffier M., Neipp M., Veres T., Frieling T., Meier P., Manns M., Bischoff S. (2006). Selective expression of histamine receptors H1R, H2R, and H4R, but not H3R, in the human intestinal tract. Gut.

[46] Schneider E.H., Seifert R. (2016). The histamine H4-receptor and the central and peripheral nervous system: A critical analysis of the literature. Neuropharmacology.

[47] Thangam E.B., Jemima E.A., Singh H., Baig M.S., Khan M., Mathias C.B., Church M.K., Saluja R. (2018). The Role of Histamine and Histamine Receptors in Mast Cell-Mediated Allergy and Inflammation: The Hunt for New Therapeutic Targets. Front. Immunol..

[48] Cataldi M., Borriello F., Granata F., Annunziato L., Marone G. (2014). Histamine receptors and antihistamines: From discovery to clinical applications. Chem. Immunol. Allergy.

[49] Deiteren A., De Man J.G., Pelckmans P.A., De Winter B.Y. (2015). Histamine H_4_ Receptors in the Gastrointestinal Tract: H4 Receptors in the Gastrointestinal Tract. Br. J. Pharmacol..

[50] Pfanzagl B., Mechtcheriakova D., Meshcheryakova A., Aberle S.W., Pfragner R., Jensen-Jarolim E. (2017). Activation of the ileal neuroendocrine tumor cell line P-STS by acetylcholine is amplified by histamine: Role of H3R and H4R. Sci. Rep..

[51] Beghdadi W., Porcherie A., Schneider B.S., Dubayle D., Peronet R., Huerre M., Watanabe T., Ohtsu H., Louis J., Mécheri S. (2008). Inhibition of histamine-mediated signaling confers significant protection against severe malaria in mouse models of disease. J. Exp. Med..

[52] Metz M., Doyle E., Bindslev-Jensen C., Watanabe T., Zuberbier T., Maurer M. (2011). Effects of Antihistamines on Innate Immune Responses to Severe Bacterial Infection in Mice. Int. Arch. Allergy Immunol..

[53] Sullivant A., Mackin A., Pharr T., Cooley J., Wills R., Archer T. (2016). Identification of histamine receptors in the canine gastrointestinal tract. Vet. Immunol. Immunopathol..

[54] Kim H., Dwyer L., Song J.H., Martin-Cano F.E., Bahney J., Peri L., Britton F.C., Sanders K.M., Koh S.D. (2011). Identification of histamine receptors and effects of histamine on murine and simian colonic excitability. Neurogastroenterol. Motil..

[55] Schirmer B., Neumann D. (2021). The Function of the Histamine H4 Receptor in Inflammatory and Inflammation-Associated Diseases of the Gut. Int. J. Mol. Sci..

[56] Scholl I., Untersmayr E., Bakos N., Roth-Walter F., Gleiss A., Boltz-Nitulescu G., Scheiner O., Jensen-Jarolim E. (2005). Antiulcer drugs promote oral sensitization and hypersensitivity to hazelnut allergens in BALB/c mice and humans. Am. J. Clin. Nutr..

[57] Anvari S., Miller J., Yeh C.-Y., Davis C.M. (2019). IgE-Mediated Food Allergy. Clin. Rev. Allergy Immunol..

[58] Raithel M., Weidenhiller M., Abel R., Baenkler H., Hahn E. (2006). Colorectal mucosal histamine release by mucosa oxygenation in comparison with other established clinical tests in patients with gastrointestinally mediated allergy. World J. Gastroenterol..

[59] Raithel M., Matek M., Baenkler H.W., Jorde W., Hahn E.G. (1995). Mucosal histamine content and histamine secretion in Crohn’s disease, ulcerative colitis and allergic enteropathy. Int. Arch. Allergy Immunol..

[60] Bijlsma P.B., Backhaus B., Weidenhiller M., Donhauser N., Hahn E.G., Raithel M. (2004). Food llergy diagnosis by detection of antigeninduced electrophysiological changes and histamine release in human intestinal biopsies during mucosa-oxygenation. Inflamm. Res..

[61] Nolte H., Schiotz P.O., Kruse A., Skov P.S. (1989). Comparison of intestinal mast-cell and basophil histamine-release in children with food allergic reactions. Allergy.

[62] Raithel M., Hagel A.F., Zopf Y., Bijlsma P.B., De Rossi T.M., Gabriel S., Weidenhiller M., Kressel J., Hahn E.G., Konturek P.C. (2012). Analysis of immediate ex vivo release of nitric oxide from human colonic mucosa in gastrointestinally mediated allergy, inflammatory bowel disease and controls. J. Physiol. Pharmacol..

[63] Nowak-Wegrzyn A., Assa’Ad A.H., Bahna S.L., Bock S.A., Sicherer S.H., Teuber S.S. (2009). Work Group report: Oral food challenge testing. J. Allergy Clin. Immunol..

[64] Ramsey N., Berin M.C. (2021). Pathogenesis of IgE-mediated food allergy and implications for future immunotherapeutics. Pediatr. Allergy Immunol..

[65] Chinthrajah R.S., Purington N., Andorf S., Long A., O’Laughlin K.L., Lyu S.C., Manohar M., Boyd S.D., Tibshirani R., Maecker H. (2019). Sustained outcomes in oral immunotherapy for peanut allergy (POISED study): A large, randomised, double-blind, placebo-controlled, phase 2 study. Lancet.

[66] Anagnostou K., Islam S., King Y., Foley L., Pasea L., Bond S., Palmer C., Deighton J., Ewan P., Clark A. (2014). Assessing the efficacy of oral immunotherapy for the desensitisation of peanut allergy in children (STOP II): A phase 2 randomised controlled trial. Lancet.

[67] (2020). Peanut Allergen Powder (Palforzia). JAMA.

[68] Kacik J., Wróblewska B., Lewicki S., Zdanowski R., Kalicki B. (2017). Serum Diamine Oxidase in Pseudoallergy in the Pediatric Population. Adv. Exp. Med. Biol..

[69] Schwelberger H.G. (2009). Histamine intolerance: Overestimated or underestimated?. Inflamm. Res..

[70] Schnedl W.J., Enko D. (2021). Histamine Intolerance Originates in the Gut. Nutrients.

[71] Schink M., Konturek P.C., Tietz E., Dieterich W., Pinzer T.C., Wirtz S., Neurath M.F., Zopf Y. (2018). Microbial patterns in patients with histamine intolerance. J. Physiol. Pharmacol..

[72] Sanchez-Perez S., Comas-Baste O., Duelo A., Veciana-Nogués M.T., Berlanga M., Latorre-Moratalla M.L., Vidal-Carou M.C. (2022). Intestinal Dysbiosis in Patients with Histamine Intolerance. Nutrients.

[73] Schwelberger H.G. (2010). Histamine intolerance: A metabolic disease?. Inflamm. Res..

[74] Dyer J., Warren K., Merlin S., Metcalfe D.D., Kaliner M. (1982). Measurement of plasma histamine: Description of an improved method and normal values. J. Allergy Clin. Immunol..

[75] Hrubisko M., Danis R., Huorka M., Wawruch M. (2021). Histamine Intolerance—The More We Know the Less We Know. A Review. Nutrients.

[76] Schnedl W.J., Schenk M., Lackner S., Enko D., Mangge H., Forster F. (2019). Diamine oxidase supplementation improves symptoms in patients with histamine intolerance. Food Sci. Biotechnol..

[77] Martin I.S.M., Brachero S., Vilar E.G. (2016). Histamine intolerance and dietary management: A complete review. Allergol. Immunopathol..

[78] Mokhtar S., Mostafa G., Taha R., Eldeep G.S.S. (2012). Effect of different starter cultures on the biogenic amines production as a critical control point in fresh fermented sausages. Eur. Food Res. Technol..

[79] Winterkamp S., Weidenhiller M., Otte P., Stolper J., Schwab D., Hahn E.G., Raithel M. (2002). Urinary Excretion of N-Methylhistamine as a Marker of Disease Activity in Inflammatory Bowel Disease. Am. J. Gastroenterol..

[80] Hagel A.F., de Rossi T., Konturek P.C., Lbrecht H., Lker S., Hahn E.G., Raithel M. (2015). Plasma histamine and tumour necrosis factor-alpha levels in Crohn’s disease and ulcerative colitis at various stages of disease. J. Physiol. Pharmacol..

[81] Smolinska S., Groeger D., Perez N.R., Schiavi E., Ferstl R., Frei R., Konieczna P., Akdis C.A., Jutel M., O’mahony L. (2016). Histamine Receptor 2 is Required to Suppress Innate Immune Responses to Bacterial Ligands in Patients with Inflammatory Bowel Disease. Inflamm. Bowel Dis..

[82] Petersen J., Raithel M., Schwelberger H.G. (2002). Histamine N-methyltransferase and diamine oxidase gene polymorphisms in patients with inflammatory and neoplastic intestinal diseases. Inflamm. Res..

[83] Schulze H.A., Hasler R., Mah N., Lu T., Nikolaus S., Costello C.M., Schreiber S. (2008). From model cell line to in vivo gene expression: Disease-related intestinal gene expression in IBD. Genes Immun..

[84] Borghini R., Donato G., Alvaro D., Picarelli A. (2017). New insights in IBS-like disorders: Pandora’s box has been opened; a review. Gastroenterol. Hepatol. Bed Bench.

[85] Mac Sharry J., O’Mahony L., Fanning A., Bairead E., Sherlock G., Tiesman J., Fulmer A., Kiely B., Dinan T.G., Shanahan F. (2008). Mucosal cytokine imbalance in irritable bowel syndrome. Scand. J. Gastroenterol..

[86] Dinan T.G., Quigley E.M.M., Ahmed S.M.M., Scully P., O’Brien S., O’Mahony L., O’Mahony S., Shanahan F., Keeling P.W.N. (2006). Hypothalamic-Pituitary-Gut Axis Dysregulation in Irritable Bowel Syndrome: Plasma Cytokines as a Potential Biomarker?. Gastroenterology.

[87] Bohn L., Störsrud S., Törnblom H., Bengtsson U., Simrén M. (2013). Self-Reported Food-Related Gastrointestinal Symptoms in IBS Are Common and Associated With More Severe Symptoms and Reduced Quality of Life. Am. J. Gastroenterol..

[88] Barbara G., Stanghellini V., De Giorgio R., Cremon C., Cottrell G.S., Santini D., Pasquinelli G., Morselli/labate A.M., Grady E.F., Bunnett N.W. (2004). Activated mast cells in proximity to colonic nerves correlate with abdominal pain in irritable bowel syndrome. Gastroenterology.

[89] Klooker T.K., Braak B., Koopman K., Welting O., Wouters M.M., Van Der Heide S., Schemann M., Bischoff S.C., Wijngaard R.M.V.D., Boeckxstaens G.E. (2010). The mast cell stabiliser ketotifen decreases visceral hypersensitivity and improves intestinal symptoms in patients with irritable bowel syndrome. Gut.

[90] Buhner S., Li Q., Vignali S., Barbara G., De Giorgio R., Stanghellini V., Cremon C., Zeller F., Langer R., Daniel H. (2009). Activation of human enteric neurons by supernatants of colonic biopsy specimens from patients with irritable bowel syndrome. Gastroenterology.

[91] Chen H., Nwe P.-K., Yang Y., Rosen C.E., Bielecka A.A., Kuchroo M., Cline G.W., Kruse A.C., Ring A.M., Crawford J.M. (2019). A Forward Chemical Genetic Screen Reveals Gut Microbiota Metabolites That Modulate Host Physiology. Cell.

[92] Barbara G., Wang B., Stanghellini V., de Giorgio R., Cremon C., Di Nardo G., Trevisani M., Campi B., Geppetti P., Tonini M. (2007). Mast cell-dependent excitation of visceral-nociceptive sensory neurons in irritable bowel syndrome. Gastroenterology.

[93] Mishima Y., Ishihara S. (2020). Molecular Mechanisms of Microbiota-Mediated Pathology in Irritable Bowel Syndrome. Int. J. Mol. Sci..

[94] Codling C., O’Mahony L., Shanahan F., Quigley E.M.M., Marchesi J.R. (2009). A Molecular Analysis of Fecal and Mucosal Bacterial Communities in Irritable Bowel Syndrome. Am. J. Dig. Dis..

[95] O’Mahony L., McCarthy J., Kelly P., Hurley G., Luo F., Chen K., O’Sullivan G.C., Kiely B., Collins J.K., Shanahan F. (2005). Lactobacillus and bifidobacterium in irritable bowel syndrome: Symptom responses and relationship to cytokine profiles. Gastroenterology.

[96] Whorwell P.J., Altringer L., Morel J., Bond Y., Charbonneau D., O’Mahony L., Kiely B., Shanahan F., Quigley E.M.M. (2006). Efficacy of an encapsulated probiotic Bifidobacterium infantis 35624 in women with irritable bowel syndrome. Am. J. Gastroenterol..

[97] Hungerford J.M. (2021). Histamine and Scombrotoxins. Toxicon.

[98] Hui J.Y., Taylor S.L. (1985). Inhibition of in vivo histamine metabolism in rats by foodborne and pharmacologic inhibitors of diamine oxidase, histamine N-methyltransferase, and monoamine oxidase. Toxicol. Appl. Pharmacol..

[99] Ijomah P., Clifford M.N., Walker R., Wright J., Hardy R., Murray C.K. (1991). The importance of endogenous histamine relative to dietary histamine in the aetiology of scombrotoxicosis. Food Addit. Contam..

[100] Siegel R., DeSantis C., Jemal A. (2014). Colorectal cancer statistics. CA Cancer J. Clin..

[101] Jess T., Simonsen J., Jørgensen K.T., Pedersen B.V., Nielsen N.M., Frisch M. (2012). Decreasing risk of colorectal cancer in patients with inflammatory bowel disease over 30 years. Gastroenterology.

[102] Herrinton L.J., Liu L., Levin T.R., Allison J.E., Lewis J.D., Velayos F. (2012). Incidence and mortality of colorectal adenocarcinoma in persons with inflammatory bowel disease from 1998 to 2010. Gastroenterology.

[103] Beaugerie L., Svrcek M., Seksik P., Bouvier A., Simon T., Allez M., Brixi H., Gornet J., Altwegg R., Beau P. (2013). CESAME Study Group: Risk of colorectal high-grade dysplasia and cancer in a prospective observational cohort of patients with inflammatory bowel disease. Gastroenterology.

[104] Louis P., Hold G.L., Flint H.J. (2014). The gut microbiota, bacterial metabolites and colorectal cancer. Nat. Rev. Microbiol..

[105] Sears C.L., Garrett W.S. (2014). Microbes, Microbiota, and Colon Cancer. Cell Host Microbe.

[106] Schwabe R.F., Jobin C. (2013). The microbiome and cancer. Nat. Rev. Cancer.

[107] Tjalsma H., Boleij A., Marchesi J.R., Dutilh B.E. (2012). A bacterial Driver–passenger model for colorectal cancer: Beyond the usual suspects. Nat. Rev. Microbiol..

[108] Arthur J.C., Perez-Chanona E., Mühlbauer M., Tomkovich S., Uronis J.M., Fan T.-J., Campbell B.J., Abujamel T., Dogan B., Rogers A.B. (2012). Intestinal Inflammation Targets Cancer-Inducing Activity of the Microbiota. Science.

[109] Arthur J.C., Gharaibeh R.Z., Mühlbauer M., Perez-Chanona E., Uronis J.M., McCafferty J., Fodor A.A., Jobin C. (2014). Microbial genomic analysis reveals the essential role of inflammation in bacteria-induced colorectal cancer. Nat. Commun..

[110] Goodwin A.C., Destefano Shields C.E., Wu S., Huso D.L., Wu X., Murray-Stewart T.R., Hacker-Prietz A., Rabizadeh S., Woster P.M., Sears C.L. (2011). Polyamine catabolism contributes to enterotoxigenic Bacteroides fragilis-induced colon tumorigenesis. Proc. Natl. Acad. Sci. USA.

[111] Rowland I.R., Weisz J., Fritz-Wolz G., Clawson G., Benedict C.M., Abendroth C., Creveling C.R. (1998). Effect of Bifidobacterium longum and inulin on gut bacterial metabolism and carcinogen-induced aberrant crypt foci in rats. Carcinogenesis.

[112] Chen C.-C., Lin W.-C., Kong M.-S., Shi H.N., Walker W.A., Lin C.-Y., Huang C.-T., Lin Y.-C., Jung S.-M., Lin T.-Y. (2012). Oral inoculation of probiotics *Lactobacillus acidophilus* NCFM suppresses tumour growth both in segmental orthotopic colon cancer and extra-intestinal tissue. Br. J. Nutr..

[113] Verma A., Shukla G. (2013). Probiotics Lactobacillus rhamnosus GG, Lactobacillus acidophilus suppresses DMH-induced procarcinogenic fecal enzymes and preneoplastic aberrant crypt foci in early colon carcinogenesis in Sprague Dawley rats. Nutr. Cancer.

[114] Cianchi F., Cortesini C., Schiavone N., Perna F., Magnelli L., Fanti E., Bani D., Messerini L., Fabbroni V., Perigli G. (2005). The role of cyclooxygenase-2 in mediating the effects of histamine on cell proliferation and vascular endothelial growth factor production in colorectal cancer. Clin. Cancer Res..

[115] Masini E., Fabbroni V., Giannini L., Vannacci A., Messerini L., Perna F., Cortesini C., Cianchi F. (2005). Histamine and histidine decarboxylase up-regulation in colorectal cancer: Correlation with tumor stage. Agents Actions.

[116] Gao C., Ganesh B.P., Shi Z., Shah R.R., Fultz R., Major A., Venable S., Lugo M., Hoch K., Chen X. (2017). Gut Microbe–Mediated Suppression of Inflammation-Associated Colon Carcinogenesis by Luminal Histamine Production. Am. J. Pathol..

[117] Yang X.D., Ai W., Asfaha S., Bhagat G., Friedman R.A., Jin G., Park H., Shykind B., Diacovo T.G., Falus A. (2011). Histamine deficiency promotes inflammation-associated carcinogenesis through reduced myeloid maturation and accumulation of CD11b+Ly6G+ immature myeloid cells. Nat. Med..

[118] Chen X., Churchill M., Nagar K.K., Tailor Y.H., Chu T., Rush B.S., Jiang Z., Wang E.B., Renz B.W., Wang H. (2015). IL-17 producing mast cells promote the expansion of myeloid-derived suppressor cells in a mouse allergy model of colorectal cancer. Oncotarget.

[119] He G.-H., Ding J.-Q., Zhang X., Xu W.-M., Lin X.-Q., Huang M.-J., Feng J., Wang P., Cai W.-K. (2018). Activation of histamine H4 receptor suppresses the proliferation and invasion of esophageal squamous cell carcinoma via both metabolism and non-metabolism signaling pathways. Klin. Wochenschr..

[120] Tanaka T., Ishikawa H. (2013). Mast cells and inflammation-associated colorectal carcinogenesis. Semin. Immunopathol..

[121] Malfettone A., Silvestris N., Saponaro C., Ranieri G., Russo A., Caruso S., Popescu O., Simone G., Paradiso A., Mangia A. (2013). High density of tryptase-positive mast cells in human colorectal cancer: A poor prognostic factor related to protease-activated receptor 2 expression. J. Cell. Mol. Med..

[122] Schirmer B., Rother T., Bruesch I., Bleich A., Werlein C., Jonigk D., Seifert R., Neumann D. (2020). Genetic Deficiency of the Histamine H4-Receptor Reduces Experimental Colorectal Carcinogenesis in Mice. Cancers.

[123] Zhang C., Xiong Y., Li J., Yang Y., Liu L., Wang W., Wang L., Li M., Fang Z. (2012). Deletion and down-regulation of HRH4 gene in gastric carcinomas: A potential correlation with tumor progression. PLoS ONE.

